# Design of Shape Memory Alloy Coil Spring Actuator for Improving Performance in Cyclic Actuation

**DOI:** 10.3390/ma11112324

**Published:** 2018-11-19

**Authors:** Je-sung Koh

**Affiliations:** Department of Mechanical Engineering, Ajou University, Suwon 16499, Korea; jskoh@ajou.ac.kr; Tel.: +82-31-219-2353

**Keywords:** shape memory alloy actuator, artificial muscle, soft robotics, soft actuator

## Abstract

Performance of the shape memory alloy (SMA) coil spring actuator in cyclic actuation as an artificial muscle is strongly related to the mechanical design of the coil geometry. This paper proposes a practical design method for improving the frequency and efficiency of the SMA coil spring actuator; by designing the SMA coil spring to have large index (coil diameter/wire diameter) and pitch angle (LIP), cooling characteristics can be improved (increasing the actuation frequency) and large deformation can be obtained. The LIP design process is based on the two-state static model that describes the displacement-force relationship of the SMA coil spring in two states—a fully austenite phase and a fully martensite phase. The design process gives accurate design parameters of the SMA coil spring actuator that satisfy the required stroke and force. The model of the fully martensite phase of the SMA coil that includes the stress-induced detwinning enables the use of maximum shear strain of the SMA. The design method reduces the mass of an SMA without changing the stroke and increase the power density and efficiency. The cyclic actuation experiments demonstrate that the LIP design doubles the maximum frequency of SMA coil actuator with one-sixth the mass of the non-LIP design.

## 1. Introduction

A shape memory alloy (SMA) actuator is widely used as an artificial muscle actuator for soft robots and small-scale robots. Compared to other types of artificial muscle actuators [1], the high-power density and ease of use have made SMA actuators attractive as a solution for lightweight and compact robots and especially for robots that are made with soft and compliant materials [2,3,4,5,6,7,8,9,10,11,12,13,14,15]. The coil-type SMA spring actuator can generate a larger displacement, above 100% of its original length, which allows it to have wider applications compared to the wire-type SMA actuator; the wire-type SMA actuator can generate a displacement less than 6–8% of its original length. The coil type can use inductance to measure the displacement without sensors [16]. The performance of coil-type SMA spring actuators is wide-ranging, in terms of force and displacement, depending on how the coil is designed [17]. Owing to the nonlinear characteristics of the SMA, the conventional coil design method will not lead to optimal performance of the SMA coil spring actuator. Therefore, it is important to design the SMA coil spring actuator based on accurate knowledge of the material properties of the SMA along with accurate understanding of how the coiling affects the behavior of the SMA actuator.

Over the last decade, SMA coil spring actuators capable of producing large strain similar to that of muscle have been applied to various bio-inspired robots as an artificial muscle, as shown in the worm-like robot [6,7,8,9,10], the micro fish fin [11], flytrap robot [12] and flea-inspired jumping robot [13]. Recently, the demand of improving power and efficiency of the actuator has increased in the soft robotics, which is the field that aims to achieve better and simpler mechanisms by exploiting the mechanical intelligence of soft materials [18]. Along with the soft robotics, the SMA actuator has increased potential utility in aspects of its high-power density and large deformation. In these applications, the conventional coil spring design has commonly been employed for the design of SMA coil springs [14,19]. The force–stroke relationships in the two SMA phases are calculated by changing the shear modulus of the SMA. Many small-scale robotic systems such as the active endoscope [15] and microrobotic fish fin [11] have used the conventional coil spring model for design of the SMA coil spring actuator. However, this model neglects one important phenomenon of the SMA—the detwinning. The martensite phase has two internal microstructure orientations, twinned and detwinned martensite. The twinned martensite changes to detwinned martensite when external stress is applied. Neglecting detwinning causes significant strain error in the SMA. S. Kim et al. [20] describe the modified coil spring model that contains the detwinning of the SMA coil spring.

As a more accurate model of SMAs, Tobushi and Tanaka [21] and Liang and Rogers [22] proposed the SMA coil spring model based on the constitutive model of the SMA developed by Tanaka [23], Liang and Rogers [24], Boyd and Lagoudas [25] and Brinson [26]. In An et al. [27], these previous models were simplified into the one-dimensional (1-D) two-state static SMA coil spring model. The two-state static model describes the loading paths, stress–strain relationships, of the SMA coil spring actuator for the fully martensite state, which represents the fully extended state and the fully austenite state, which represents the fully contracted state. The behavior of the SMA in these two states is sufficient to design the actuator; the actuation stroke for a given loading condition can be found from the difference in the strain between the two states. The design that maximizes the strain, maximizes the efficiency of the SMA coil spring actuator. 

**Figure 1:**
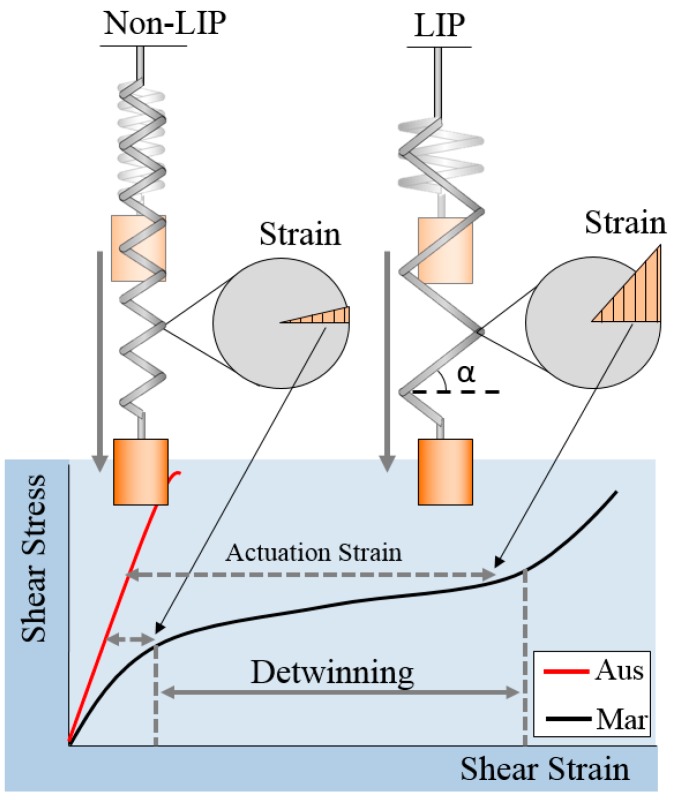
Comparison between large index and pitch angle (LIP) design and non-LIP design of shape memory alloy (SMA) coil spring actuators. The LIP design has a large pitch angle (*α*) and shear strain that can use the detwinning region of the SMAs. The large coil diameter improves the cooling performance for high-frequency actuation. The graph shows the representative shear stress–shear strain relationship in Austenite phase (Aus) and Martensite phase (Mar).

In this paper, a new method for designing the SMA coil spring actuator that uses the two-state static model is proposed for improving the frequency and efficiency in cyclic actuation. It is a practical method for designing an efficient and robust SMA coil spring actuator. The design is based on the concept that the SMA coils should have a large index and pitch angle (LIP) in order to use the unique characteristics of the SMAs. The LIP design allows for the use of the large strain region by detwinning, as shown in Figure 1. The LIP SMA spring is shown to have a large pitch angle and deform much more than the non-LIP coil spring. This design maximizes the efficiency of the SMA coil spring actuator because the actuation strain is maximized and it can save the length of the SMA wire usage in same stroke and force. A proof of concept study of the LIP design of the SMA coil spring actuator showed improved frequency and efficiency in a small-scale robot system [8]. Many other methods have been applied to improve the performance and efficiency of the SMA actuators, including the use of coolant, alloy composition design, heat treatments as well as different geometrical and mechanical designs. Wet SMA actuators [28,29] exhibit improved efficiency by immersing the SMA wire in a fluid such as a coolant. The heat treatment or the material composition changes the transition temperature, which improves the frequency but a linear relationship between those has not been fully developed [30]. In micro scale, the SMA actuator can achieve a high frequency up to kHz by the high convection-cooling rate and the resonance of the actuator [31,32,33]. These approach would be suitable for micro scale systems that have a small payload requirement. The LIP design method presented in this paper is an approach to improve the actuation frequency of SMA coil actuator that achieves the desired force and stroke by optimizing the mechanical design of the coil in general. 

In the following section, we describe the dominant characteristics of the SMA coil spring actuator that affect the design and the actuation performance. In Section 3, the static two-state 1-D model of the SMA coil spring actuator is developed by modifying the previously proposed model and evaluated by comparison with experimental results. Using the model, the design method for the SMA coil spring actuator is described in detail. Finally, the actuation experiment results show the improvements in the SMA coil spring actuator in terms of frequency and efficiency.

## 2. Characteristics of the LIP SMA Coil Spring Actuators 

For the comprehensive and accurate design of the SMA coil spring actuator, this section describes the key material properties of the SMA used as a coil spring actuator. The fundamental characteristic of the SMA actuator is that it extends with detwinning in the martensite phase at low temperature and contracts in the austenite phase at high temperature. Therefore, the detwinning strain is a key parameter of the SMA actuator and the coupled characteristics related with the temperature are simplified into two states; fully austenite and fully martensite. Furthermore, how the coil geometry affects the actuation behavior and thermal convection characteristics should be considered.

The SMA in the martensite phase has two crystal structure orientations [34]; twinned martensite, which is the initial form after transforming from austenite and detwinned martensite, which is the deformed structure following the application of stress. The transition process is called detwinning as shown in Figure 1. In the detwinning process, the SMA creates a large displacement. When the SMA is heated from the detwinned martensite phase, the crystal structures transform back to the austenite phase, returning to its original shape. The SMA wire actuator produces the actuation force and stroke during this transformation process and the force and the stroke are calculated directly from the stress–strain relationship of the SMA materials with the dimensions of the wire (diameter, length).

The dominant deformation of the SMA coil spring, unlike the wire type, occurs in the shear mode instead of the tensile mode, as shown in the formula for the mechanical coil spring, *F* = π*d*^3^*Gγ*/8*D*, where F is the force, d is the wire diameter, *G* is the shear modulus of the material, *γ* is the shear strain, *D* is the coil diameter. Force and displacement are obtained by calculating the geometrical equation of the coil spring with the shear modulus [15]. Therefore, the shear stress and shear strain relationship should be used in designing the coil spring actuator in order to utilize the unique characteristics of the SMA, such as detwinning. In the following section, the model of the SMA coil spring actuator is developed based on the properties obtained from the shear stress and shear strain relationship. The shear strain is represented using the coil pitch angle (*α*) in the final model equation.

Generally, the frequency of the SMA actuator is determined by the heat transfer characteristics, which are related to the geometrical shape and environmental conditions. A wire with a smaller diameter cools faster because of the lower heat capacity, which is proportional to the mass. Regarding the coil, loose coils cool fast because they have better convection. Therefore, the coil index (*D*/*d*), which is the coil diameter divided by the wire diameter, is an important parameter to maximize the frequency of the SMA coil spring actuator. A higher coil index is better for increasing the maximum frequency.

The design procedure for optimizing the efficiency of the SMA coil spring actuator is based on these two principles; a large pitch angle to use the full detwinning region for reducing mass and a large coil index for improving the frequency.

## 3. Static Two-State Model of the SMA Coil Spring Actuator 

The nonlinear behavior of the SMA makes modeling difficult. The detwinning of the SMA allows the coils to elongate much more than the coils made with other metallic materials. A 1-D model of the SMA coil spring actuator was developed previously by S. An et al. [27]. This model describes the force-displacement relationship of the SMA coil spring in two states; fully austenite and fully martensite. These two extreme states are enough for determining the SMA coil spring design parameters to achieve desired force and stroke. The model basically has two equations; one for the fully austenite phase and another for the fully martensite phase. Both equations contain the geometrical nonlinearity caused by large deformation of the coil. In the fully martensite equation, the detwinning behavior of the SMA coil spring actuator is included. However, it requires a numerical technique to compute the force–displacement relationship because of a recursive term in the formulation. Herein, the recursive term is modified as follows, which makes the model easier to use.

The force–displacement relationship is expressed as Equations (1) and (2) at the austenite and martensite phases, respectively. They are derived by combining the conventional coil spring equation and the large deformation term. Additionally, Equation (2) has a detwinning term, which is the function of the detwinned martensite volume fraction (*ξ_Sγ_*):

(1) $$F_{A}=\frac{G_{A}d^{4}}{8D^{3}n}\left(\frac{\cos^{3}\alpha_{i}}{\cos^{2}\alpha_{Af}\left(\cos^{2}\alpha_{Af}+sin^{2}\alpha_{Af}/\left(1+\nu \right)\right)}\right)\delta_{A}$$

(2) $$F_{M}=\frac{G_{M}d^{4}}{8D^{3}n}\left(\frac{\cos^{3}\alpha_{i}}{\cos^{2}\alpha_{Mf}\left(\cos^{2}\alpha_{Mf}+sin^{2}\alpha_{Mf}/\left(1+\nu \right)\right)}\right)\delta_{M}-\frac{\pi d^{3}}{8D}G_{M}\gamma_{L}\xi_{S\gamma }$$

The material properties are: *G*, the shear modulus, *ν*, the Poisson’s ratio and *γ_L_*_,_ the residual strain. The material properties can be obtained from tensile test of SMA. The design parameters that should be determined by the user are: *d*, the wire diameter, *D*, the coil diameter, *α_i_*, the initial pitch angle and *n*, the number of the coils. The variables are: *F*, the force, *ξ_Sγ_*, the detwinned martensite volume fraction, that changes with temperature and stress, *δ*, the displacement and α_f_, the final pitch angle. Subscripts *A* and *M* denote the austenite and the martensite, respectively. 

The deformation is represented with different variables to improve the understanding of the equations. The displacement *δ* and the final pitch angle *α_f_* are different representations of the deformation, along with the shear strain *γ* in Equation (5). Although these three variables are dependent variables, combining these variables complicates the equations; we use the final pitch angle in the term that describes the large deformation of the spring, the displacement for the original spring force-displacement equation and the shear strain for the detwinning term. We provide equations that describes the relationship of these three variables so that they can be converted as needed. The final pitch angle can be converted to the displacement of the coil spring with Equation (3). The final pitch angle can be converted to the shear stain (*γ*) with Equation (4):

(3) $$\delta =\frac{\pi nD}{\cos\alpha_{i}}\left(\sin\alpha_{f}-\sin\alpha_{i}\right)$$

(4) $$\gamma =\frac{d}{D}\frac{\cos^{2}\alpha_{i}\left(\sin\alpha_{f}+sin\alpha_{i}\right)}{\cos^{2}\alpha_{f}\left(\cos^{2}\alpha_{f}+sin^{2}\alpha_{f}/\left(1+\nu \right)\right)}$$

The force can be converted to the shear stress (*τ*) with Equation (5):

(5) $$\tau =\frac{8DF}{\pi d^{3}}$$

With these conversion equations, the force-displacement relationship can be converted to shear stress-shear strain relationship. Theoretically, a single shear stress-shear strain relationship can be implemented into coil springs with various force-displacement relationships. 

The detwinned martensite volume fraction (*ξ_Sγ_*) is commonly a function of shear stress (*τ*) and temperature. In our model, we assume that the temperature is low enough to maintain fully martensite phase. The shear stress (*τ*) is a function of the force (*F*), as shown in Equation (5). Therefore, the force (*F*) and the detwinned martensite volume fraction (*ξ_Sγ_*) become recursive in Equation (2). This recursive relationship requires a computational technique to plot the model, such as updating the previous step data in the numerical computation. Furthermore, it becomes easily unstable when the step interval is selected improperly and the gap between the detwinning start and finish stress, that is, the plateau region of the martensite loading path, is too narrow to distinguish by stress in the modeling. To avoid these instability problems and complex calculations, the function of the detwinned martensite volume fraction (*ξ_Sγ_*) is modified to be a function of the shear strain (*γ*) as follows:

(6) $$\xi_{s\gamma }=\frac{1}{2}\cos\left(\frac{\pi }{\gamma_{s}^{cr}-\gamma_{f}^{cr}}\left(\gamma -\gamma_{f}^{cr}\right)\right)$$

where *γ^cr^* is critical strain for detwinning start ($\gamma_{s}^{cr}$) and finish ($\gamma_{f}^{cr}$), which is a material properties of SMA can be obtained by tensile test.

Therefore, Equation (2) is no longer recursive by using Equation (6).

To summarize, Equations (1) and (3) are used to plot the force-displacement relationship for the fully asustenite phase and Equations (2)–(4) and (6) are used to plot the force-displacement relationship for the fully martensite phase. Equations (3)–(5) are used to convert the force-displacement relationship to a shear stress, shear strain relationship; the force-displacement relationship can be acquired from tensile tests of a SMA coil spring. For example, if the displacement of the coil spring is determined, α can be obtained from Equation (3) and *γ* and *ξ_S_*_γ_ can be obtained from Equations (4) and (6). Finally, the force is computed from Equations (1) and (2) in the austenite and martensite phases, respectively. Therefore, we can obtain the force-displacement relationship and the shear stress-shear strain relationship for the two phases.

## 4. Validation of the Static Two-State SMA Coil Spring Model 

Three types of SMA coil spring actuators are prepared to obtain material properties such as the shear modulus (*G*), residual strain (*γ_L_*) and critical strains (*γ^cr^*) for verifying and completing the model equations. The SMA coil spring actuator is fabricated by winding the SMA wire (Dynalloy Co., Irvine, CA, USA) around the core wire and curing in the furnace. The different design parameters of the three SMA coil springs are listed in Table 1. The initial pitch angle, *α_i_*, is designed to be zero. Tensile experiments are performed with the tensile test machine with an oven chamber. The force and the displacement of the three SMA coil springs are measured in different temperatures. SMA properties are obtained from the experimental results and are listed in Table 2. The shear modulus is the slope of the shear stress–shear strain graph. The slope of the graph for the austenite phase is almost constant and the modulus (*G_A_*) can be obtained by a first-order fitting line but the graph for the martensite phase shows nonlinear behavior. The modulus of the martensite phase (*G_M_*) is the slope of the initial linear region of strain in 1%. The residual strain indicates the strain width of the plateau region by detwinning. Detwinning start and finish critical strains are used for the function of the detwinned martensite fraction, as shown in Equation (6).

**Figure 2:**
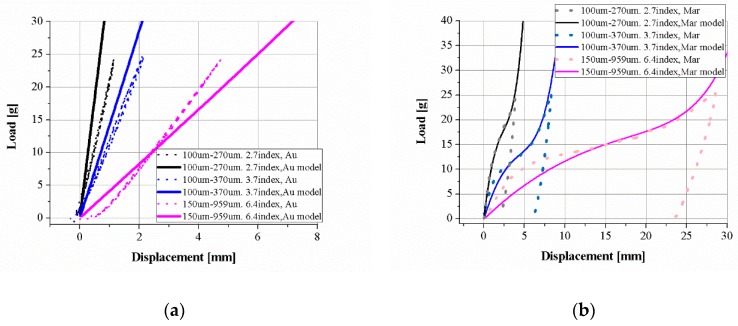
Comparison of experimental results with the static two-state model. Tensile test results and model plot of three kinds of SMA coil spring: (**a**) the austenite phase at high temperature and (**b**) the martensite phase at room temperature.

Figure 2 shows the experimental results and model plot of the three SMA coil spring samples. The model plots with the material properties obtained from experiments match the experimental data well for various coil parameters. The austenite phase plots have little more variation than the martensite plots because the SMA is very sensitive to temperature and it is hard to guarantee a constant temperature in various experiments, as shown in Figure 2a.

**Figure 3:**
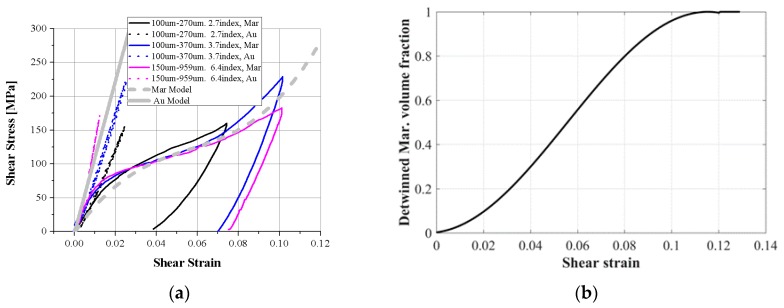
(**a**) The strain–stress relationship converted from displacement–force results of three kinds of SMA coil spring; (**b**) the detwinned martensite volume fraction of the martensite model.

The stress–strain relationship is obtained by converting the force–displacement relationship using Equations (4) and (5), as shown in Figure 3a. The gray solid line is converted from the model of the austenite phase and the three model graphs are converted to a single shear stress–shear strain graph. As described above, the three experimental graphs have little variation. The gray dotted line is the martensite graph. The three experimental graphs match the model well. Figure 3b is the detwinned martensite volume fraction in the model. Detwinning starts with 1% deformation and ends at approximately 12% shear strain; these critical values are obtained from the experimental results.

From the experimental results, we propose the SMA coil spring actuator to be designed to use 8–12% of the maximum shear strain in order to use the full detwinning region of the shear strain. Depending on applications, we decide the maximum shear strain how much the SMA coil spring actuator is released. In case that requires high actuation cycle number, 8% is proper because the smaller maximum strain may reduce degradation of the performance in usage. In case that requires high power and speed, extremely 12% can be used in the design process.

## 5. LIP SMA Coil Spring Design Procedure

Using the static two-state SMA coil spring model, we can design the SMA coil spring actuator that produces the desired force and displacement. The design procedure starts with selecting the minimum wire diameter “*d*”. The wire diameter “*d*” is a dominant design parameter of the SMA coil spring actuator and is the most sensitive parameter to the force–displacement relationship and cooling performance related to the actuation frequency. The minimum wire diameter leads to the shortest cooling time and the lowest material mass of the LIP SMA coil spring actuator. The initial value is the minimum diameter available among the off-the-shelf SMA wires.

After selecting the wire diameter “*d*”, the maximum shear strain should be determined according to the actuation cycle number before failure. Commonly, 6% axial stain is recommended by manufacturers but SMA recovers its original length from much more extension than 6% in multiple experiments. From the results of a shear strain recovery test based on the model, greater than 12% shear stain can recover to the original shear strain within tens of actuation cycles and 8% shear strain shows hundreds-fold recovery without permanent deformation. Based on this background, we set the maximum shear strain range of the LIP design as 8–12%. The 8% maximum shear strain is for the robust actuator that has a long life cycle but uses little more material and the 12% maximum shear strain is for the fastest and lowest mass actuator that has a short life cycle. The main purpose of the design procedure is to use the full strain range of the SMA.

The procedure for computing a coil diameter “*D*” is an iteration of model Equations (2)–(4) as “*D*” increases gradually. The initial value is set to be a bit higher than the wire diameter “*d*”, and the force is computed when the shear strain reaches the determined value in advance. If “*D*” is small, the force exceeds the desired value before the shear stain reaches the maximum value. In that case, the computation is repeated with increasing “*D*”. While increasing “*D*”, if the force meets the required value at the maximum shear strain, the iteration stops and the “*D*” value at the final step is the maximum coil diameter. After computing the maximum “*D*” with the minimum “*d*”, the coil index “*D*/*d*” should be checked for verifying manufacturability. Practically, it is impossible to make the coil if the coil index “*C* = *D*/*d*” is below 3. We set the minimum coil index to 4, adding the safety factor. If the coil has an index value lower than 4, it can be increased by the larger wire diameter “*d*”. With a larger “*d*”, the coil diameter “*D*” is computed again by the above procedure. If the coil index is higher than 4, the single coil design is finished. The single coil stroke with the desired load is determined at this step. The next step is computing the coil number “n.”

The coil number “*n*” is computed with the desired stroke and the single coil stroke at the required loading condition. The single coil stroke is obtained from the displacement gap between the martensite and austenite models from Equations (1) and (2). The coil number “*n*” is the value of the desired actuation stroke divided by the single coil stroke. Finally, we obtain all design parameters of the coil (*d*, *D*, *n*) that can use the entire strain range of the detwinned martensite. 

## 6. Actuation Performance Experiments of the LIP SMA Coil Spring Actuator

Two SMA coil spring actuators which have different design parameters but same maximum force and stroke are prepared to compare the actuation performance of LIP and non-LIP SMA coil spring actuators. The two actuators are designed by the two-state static model and have nearly the same stroke on the same loading condition. Using the LIP design process, an LIP SMA coil spring actuator is designed to have 8% of the maximum shear strain in extension at the martensite phase. The load is 0.5 N and the stroke is measured as 50 mm. Actually, it is designed to have a stroke of 40 mm at 0.5 N load but the dynamic extension of the actuator increases the stroke to 50 mm because experiments are performed as fast cyclic actuation and the inertia of the weight effects the actuation stroke. Non-LIP coil spring actuators have a smaller coil diameter and much larger coil number than LIP coil spring actuators in order to match the stroke at the same load with LIP SMA coil spring actuators.

The design of the LIP SMA coil spring actuator follows the design procedure described in Section 5. The wire diameter is set as 150 μm at first and the coil diameter is computed at 410 μm. The resultant coil index is 2.73, which is hard to fabricate, so the wire diameter is increased to 250 μm. The coil diameter is then computed as 1.86 mm and the coil index is 6.4. This is the largest coil index, so the coil spring could extend to be the maximum 8% shear strain on the 0.5 N loading. It is difficult, however, to make the coil diameter exactly 1.86 mm in house, so the diameter is changed to 1.75 mm coil, which can be produced by winding the 0.25 mm wire around a 1.5 mm core wire. The 0.25 mm SMA wire is wound around the 1.5 mm core wire and becomes the 1.75 mm coil spring after 400 °C annealing in a furnace. The single coil stroke is calculated as 2 mm using the displacement data from Equations (1) and (2) and the maximum pitch angle is 21.3°. Finally, the coil number is calculated by dividing the required stroke by the single coil stroke. In this case, the coil number is 20.

**Figure 4:**
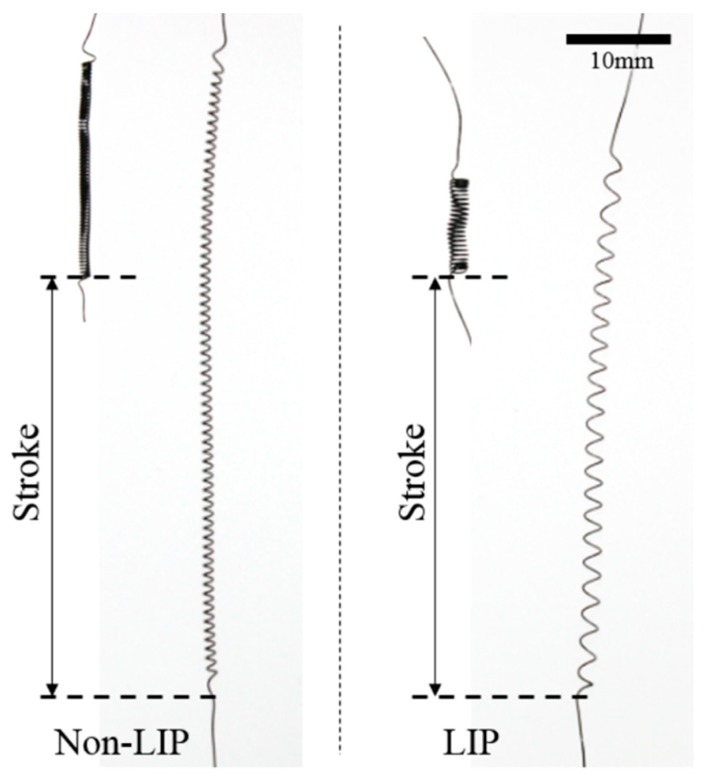
Fabricated SMA coil spring actuators; non-LIP SMA coil spring actuator (left).

The non-LIP SMA coil spring actuator is designed to have the same stroke as the LIP SMA coil spring actuator at 0.5 N loading. The wire diameter is identical to that for the LIP SMA coil spring actuator but the coil diameter and the coil index are smaller. The single coil stroke of the non-LIP SMA coil spring is much smaller than that of the LIP SMA coil spring. Therefore, it requires a much larger number of coils in order to produce the same stroke as the LIP SMA coil spring actuator. The non-LIP SMA coil spring actuator has 66 coils and a 40 mm stroke with the 3% maximum shear strain and 11.7° maximum pitch angle. 3% is too small to use the detwinned martensite region and the following experimental results show that it is an inefficient design for an SMA coil spring actuator. Figure 4 shows two SMA coil spring actuators made by winding the SMA wire around the core wire and curing in the furnace at 400 °C. The two actuators are designed to have the same stroke at the same load, although they have different coil parameters. The design parameters of these two SMA coil spring actuators are listed in Table 3.

**Figure 5:**
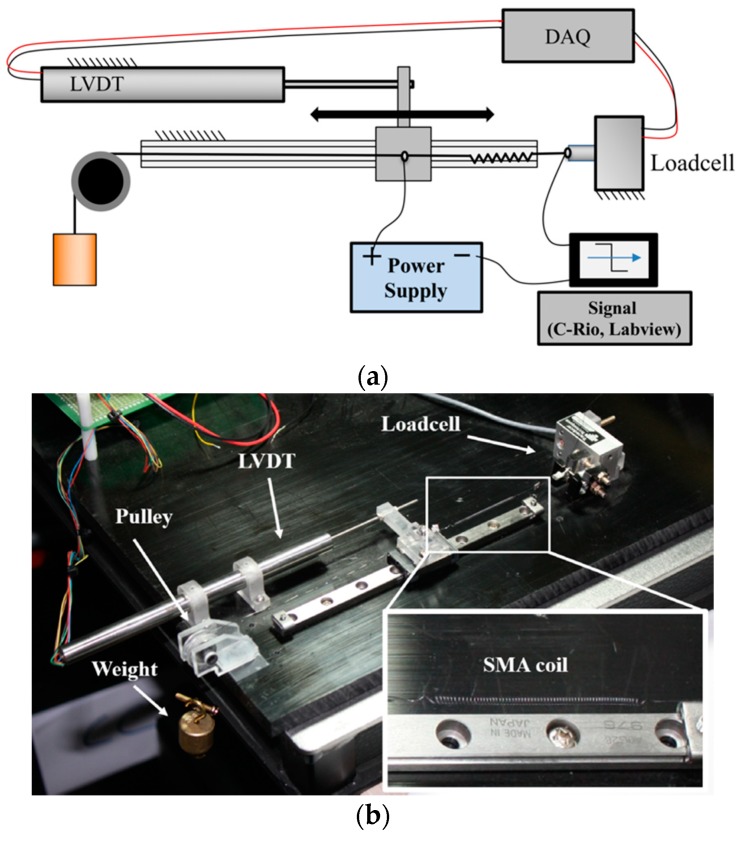
(**a**) Schematic diagram of the cyclic actuation experimental setup; (**b**) actual experimental setup.

The performance experiments are performed as cyclic actuation under constant loading conditions. Figure 5a is a schematic diagram of the experimental setup. The SMA coil springs are attached between the loadcell and linear guide. The force and displacement of the SMA coil spring are measured using the loadcell and the linear variable differential transformer (LVDT) displacement sensor of the linear guide. Figure 5b is the actual experimental environment. The parts are made of high-strength polycarbonate by the computerized numerical control (CNC) machining. The loadcell has a 500 g capacity (GSO-500, Transducer Tech. Co., Temecula, CA, USA). The LVDT (M-12 50, Measurement Specialties Inc., Hampton, NH, USA) and the ball bearing linear guide minimize the friction. A 50 g weight is hung at the end of the SMA coil spring.

**Figure 6:**
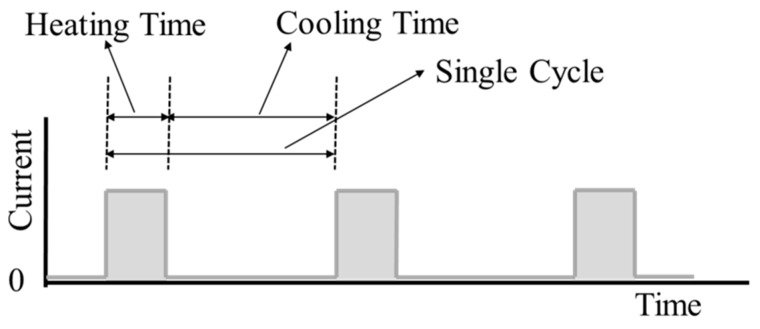
Input signal description.

**Figure 7:**
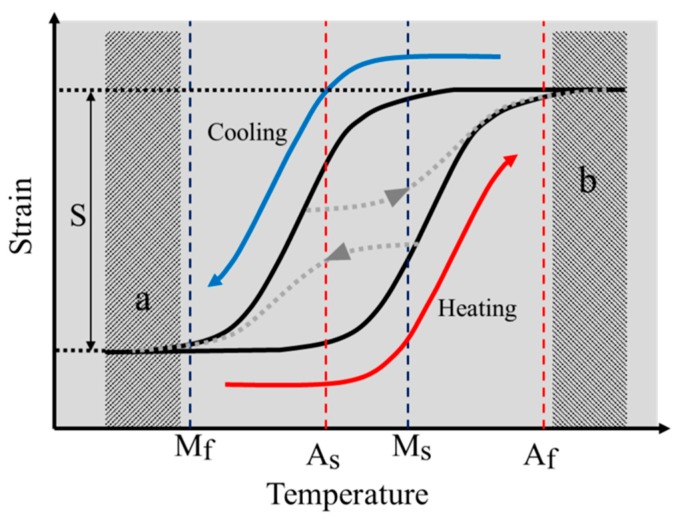
The typical Temperature-Strain relationship of the SMA actuators.

The SMA coils are actuated using Joule heating by applying an electric current. In order to find the maximum actuation frequency, actuation experiments are performed with various periods and duty ratios of electric current. A single actuation cycle has heating time and cooling time, as shown in Figure 6. During the heating time, electric current is applied to contract the SMA coil spring actuator. During the cooling time, the electric current is cut off and the actuator extends. In order to make use of the full stroke of the SMA actuator, the heating time should be long enough to heat above the Austenite finish temperature, *A_f_* and the cooling time should be long enough to cool below the Martensite finish temperature, *M_f_*. On the other hand, the heating and the cooling time should not be longer than these required times for maximum efficiency. Figure 7 shows the temperature and strain relationship on the constant loading condition. This graph presents the hysteresis curve of SMA in heating and cooling cycle. The full stroke, *S*, can be achieved by heating the SMA above *A_f_* and cooling it below *M_f_*. In terms of the actuation frequency, the maximum frequency in cyclic actuation with a designed full stroke can be achieved by following the heating (lower arrow) and cooling (upper arrow) path of the temperature-strain graph in Figure 7 that has enough cooling and heating time and no redundant heating and cooling. If the heating time is too short, the stroke becomes shorter as the lower dotted line path. If the cooling time is too short, the stroke becomes shorter as the upper dotted line path. In contrast, too much heating time or too much cooling time causes redundant heating as region ‘b’ or redundant cooling as region ‘a’. Different coil actuators have different minimum heating and cooling time for maximum frequency in cyclic actuation.

To find the minimum time that can produce the desired stroke, we perform the experiments with the practical set-up. Theoretically, the applied electric energy (*Q_E_*) is converted to the Joule heating of SMA coil spring (*Q_J_*), the convection (*Q_c_*) and latent heat for transformation (*Q_L_*) for heating. The energy conservation takes form:

*Q_E_* = *Q_J_* + *Q_c_* + *Q_L_* (7)

The applied electric energy (*Q_E_*) is computed by product of the applied voltage (*V*), current (*I*) and time (*t*):

*Q_E_* = *VIt* (8)

The Joule heating (*Q_J_*) is the energy that increase temperature of the SMA expressed as:

*Q_J_* = *m*C(*T_f_* − *T_i_*) (9)

where *m* is mass, C is specific heat and *T* is the temperature and the subscript *f* and *i* indicate the final and the initial temperature. *Q_c_* is the convection energy that flows away while heating and is expressed as:

*Q_c_* = *hA*(*T*_∞_ − *T_SMA_*(*t*))*dt* (10)

where *h* is the convection coefficient, *A* is the surface area, *T_∞_* is the environment temperature and *T_SMA_* is the surface temperature of the SMA. *Q_L_* is the latent heat for the phase transformation of the SMA from detwinned Martensite to Austenite and is expressed as:

*Q_L_* = *mL* (11)

where *m* is the mass and *L* is the specific latent heat. If the electric power is high enough to achieve a short activation time, we can ignore the convection term in the heating process. Then, the electric energy will be used for increasing the temperature and the phase transformation of SMA. The specific heat (C) and specific latent heat (*L*) are about the order of 10^2^ (J/kg K) and 10^4^ (J/kg), respectively [35]. Assuming that the specific heat for increasing temperature is the order of 10, the latent heat for phase transformation is the order of 10^3^ (J/kg). Hence, we can suppose that the latent heat for phase transformation takes more energy from the applied electric energy than the heat for increasing temperature. We can quantify the amount of energy by measuring the heating time. In experiment, the minimum heating time means the required energy for heating the SMA coil actuator above the transition temperature. 

In the cooling process, the SMA actuator is cooled by the convection and the latent heat for phase transformation from Austenite to Martensite. In the heating process, we quantify the cooling characteristics of the SMA coil spring actuator by measuring the minimum cooling time. The minimum cooling time would represent the convection characteristics of the SMA coil actuator, which is the time-dependent factor.

With this experiment, we obtain the minimum heating and cooling time, the maximum frequency and the applied electric energy of the SMA coil spring actuators which have different design parameters and compare these parameters to show how much the LIP design improves the frequency and efficiency with full stroke by reducing the heating time, the cooling time and the applied electric energy.

## 7. Results

**Figure 8:**
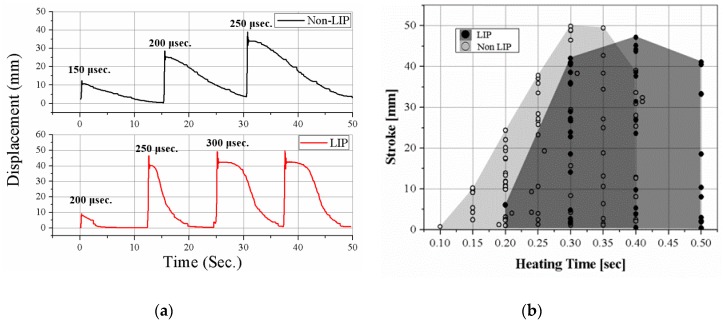
Cyclic actuation results for various heating times: (**a**) time–displacement responses for increasing heating time; (**b**) actuation stroke results depending on heating time.

To measure the minimum heating time that can follow the red path in Figure 7, we increase the heating time over 50 ms starting from 100 ms for each experiment. The stroke is measured when the cyclic actuation becomes stable without overheating and overload in the actuation condition. Insufficient heating time causes a short stroke because the SMA is not sufficiently heated. Figure 8a shows the actuation displacement graph of results when heating time is insufficient. The cooling time is enough to release the SMA but the actuation stroke is much shorter than the full stroke, 40 mm, as the first and second actuations in the graph for the non-LIP SMA coil and the first actuation in the graph for the LIP SMA coil. The minimum heating times of the two SMAs are negligibly different around 300 to 400 ms, as shown in Figure 8b. The heating time is comparatively very short and the effect of thermal convection around the coil is small during the heating. Therefore, the heating times of the two coils are similar but the LIP SMA coil requires a little more heating time because of its high convection property and the latent heat for transformation. Theoretically, required heating times without the convection effect of the LIP and the non-LIP SMA coil are 130 milli-seconds and 500 milli-seconds (*t* = *m*C*ΔT*/*VI*, where C is the specific heat of SMA, 0.84 J/g °C, *T* is the temperature, *V* is the electric voltage, *I* is the electric current). We can find that the LIP SMA coil have high convection characteristics which cause more time than theoretical heating time. Furthermore, the LIP SMA coil has more detwinned martensite volume fraction that may require more latent heat for transforming from detwinned Martensite to Austenite phase. However, it does not cause the significant change in the final actuation frequency.

**Figure 9:**
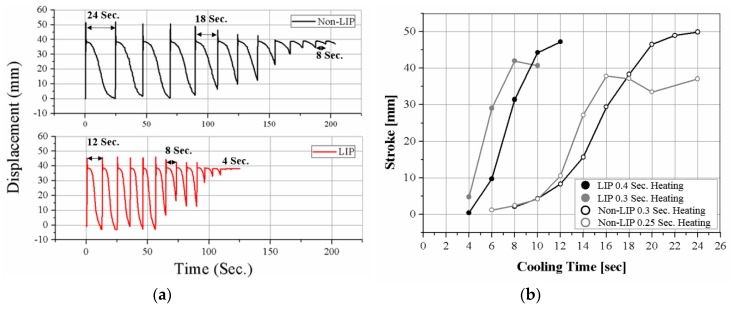
Cyclic actuation results for various cooling times: (**a**) time–displacement responses for decreasing cooling time; (**b**) actuation stroke results depending on cooling time with two different heating times; insufficient heating time (gray lines), optimal heating time (black lines).

The cooling times of two SMAs are quite different, as shown in Figure 9. Figure 9a shows the actuation displacement as the cooling time decreases. The stroke decreases as the cooling time decreases. If the cooling time is insufficient, the SMA coils do not return to the original length, as shown in Figure 9a. The LIP SMA coil spring actuator cools in 12 s, while the non-LIP requires 24 s to achieve the full stroke. The LIP coil is approximately two times faster than the non-LIP coil. In Figure 9b, the black lines of the graph are the stroke with sufficient heating time and the gray lines are the stroke with insufficient heating time for comparing the effects of the heating time and the cooling time. The SMAs under insufficient heating cool slightly faster but they do not produce the full stroke of the actuation, as shown in Figure 9b. These experimental results indicate that the cooling characteristic of the SMA coils is a dominant factor for determining the actuation frequency, unless the coil is used in a specific environment such as in coolants.

**Figure 10:**
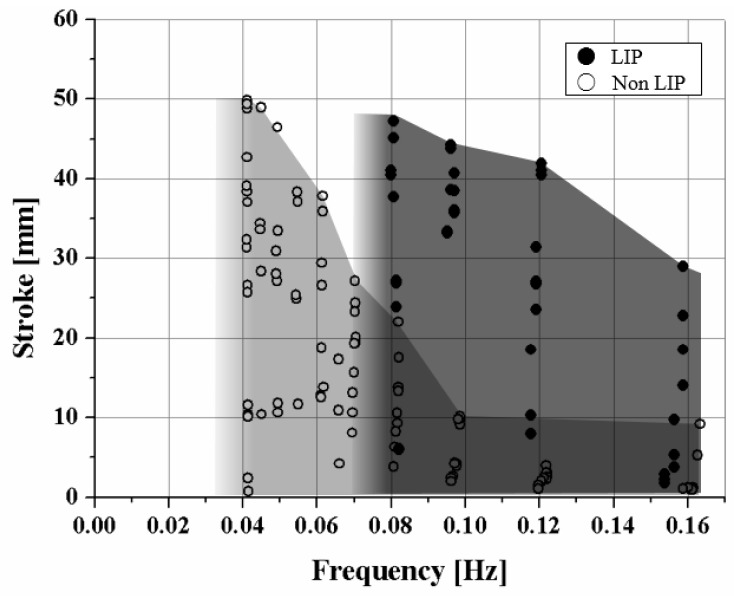
Stroke versus frequency graph merging the experiments with various heating and cooling times. The graph shows the maximum frequency of two SMA coil spring actuators that can produce a full stroke.

The maximum frequency difference of the two SMA coils is presented well in the frequency–stroke domain, as shown in Figure 10. The maximum frequency of the LIP coil is two times higher than that of the non-LIP coil, resulting from the cooling time. The stroke of the non-LIP coil starts to decrease when the frequency increases to 1/25 Hz. However, the LIP coil maintains the full stroke to a frequency of 1/12.5 Hz, which is two times higher than that of the non-LIP coil.

The experimental results for the two coil designs are listed in Table 4. The power of the LIP SMA coil spring actuator is 2 mW and that of the non-LIP SMA coil spring actuator is 1 mW. However, the mass of the LIP coil (41 mg) is 6 times heavier than that of the non-LIP coil (249 mg). The low mass of the LIP coil requires less electric power for Joules heating. Specifically, the LIP coil requires one-sixth the power that the non-LIP coil requires for heating above the transformation temperature. The power density of the LIP coil (48.78 mW/g) is 12 times higher than that of the non-LIP coil (4.01 mW/g), which means that the LIP coil produces 12 times more power with same mass of materials than the non-LIP coil. The LIP SMA coil spring actuator uses a much larger strain range than the non-LIP coil spring actuator. Therefore, the LIP produces the same stroke with much less SMA material. The LIP coil spring design process enables the use of the full stress-induced detwinning region that can derive large deformation of the SMA material.

## 8. Conclusions

The design framework and modeling provide a clear guideline for the design of the SMA coil spring actuator as an artificial muscle for the soft robot which requires large deformation of the body structure and the actuator. A new practical design methodology for the SMA coil spring actuator is proposed in terms of mechanical design, the wire diameter, the coil diameter and the coil number. The large index and pitch angle (LIP) design maximizes the frequency by improving cooling performance and the efficiency by reducing the mass of the material and input energy. The LIP design method enables production of the required actuation stroke under specific loading conditions with minimum material and maximum frequency. In this paper, the LIP design improved frequency by a factor of 2 and power density by a factor of 12 compared to the non-LIP design but these values are the results from this specific case study and various design of SMA coil spring actuators can be optimized for achieving maximum power density.

The static two-state model of the SMA coil spring is modified in order to optimize the strain range of the SMA. The proposed model describes the nonlinear tensile behavior of the SMA coil spring actuator with material properties obtained by the experiment. Various coils with different parameters match the model simulation well. With the two-state model, the design process is established with two main principles: largest index and pitch angle. The cyclic actuation tests verify the improvement of the SMA coil spring actuators in frequency, mass and efficiency by comparing non-LIP and LIP designs. Therefore, when using SMA coil actuator, the LIP design should be first considered before applying various control methods or extra devices to improve the efficiency and performance. The practical design method and the 1-D model can be easily integrated with soft robotic mechanisms that has their own compliant characteristics.

## Figures and Tables

**Table 1:** Design parameters of the SMA coil spring samples.

Parameters	Coil 1	Coil 2	Coil 3
Wire Diameter (*d*) [μm]	100	100	150
Coil Diameter (*D*) [μm]	270	370	959
Coil Number (*n*)	20	20	20
Coil Index (*D*/*d*)	2.7	3.7	6.4

**Table 2:** Material properties of SMA.

Properties	Unit	Value
Shear Modulus in Austenite (*G_A_*)	GPa	11.26
Shear Modulus in Martensite (*G_M_*)	GPa	4.7
Residual Strain (*γ_L_*)	%	6
Detwinning Start Critical Strain (*γ_s_^cr^*)	%	1
Detwinning Finish Critical Strain (*γ_f_^cr^*)	%	12

**Table 3:** Design parameters of two SMA coil spring actuators for comparison of LIP and non-LIP (0.5 N load, 40 mm stroke).

Parameters	LIP	Non-LIP
Wire Diameter (*d*) [μm]	250	250
Coil Diameter (*D*) [μm]	1750 (1860)	950
Coil Number (*n*)	20	66
Coil Index (*D*/*d*)	6.4	3.7
Pitch Angle (*α_f_*) [°]	21.3	11.7
Max. Strain	8%	3%
Mass [mg]	41	249

**Table 4:** Experimental Results of LIP and Non-LIP SMA coil spring actuators.

Properties	LIP	Non-LIP
Load [N]	0.5	0.5
Max. Stroke [mm]	48	50
Max. Frequency [Hz]	0.08	0.04
Power [mW]	2	1
Power Density [mW/g]	48.78	4.01
Input Electric Power	13.3 W (7 V 1.9 A)	19 W (10 V 1.9 A)
